# Validity and Reliability of a Self-administered Questionnaire for 24-hour Movement Behaviors

**DOI:** 10.2188/jea.JE20250185

**Published:** 2026-04-05

**Authors:** Aino Kitayama, Kaori Ishii, Ai Shibata, Akitomo Yasunaga, Bronwyn Clark, Neville Owen, David W. Dunstan, Koichiro Oka

**Affiliations:** 1 Graduate School of Sport Sciences, Waseda University, Saitama, Japan; 2 Faculty of Sport Sciences, Waseda University, Saitama, Japan; 3 Institute of Health and Sport Sciences, University of Tsukuba, Ibaraki, Japan; 4 Faculty of Health Sciences, Aomori University of Health and Welfare, Aomori, Japan; 5 School of Human Movement and Nutrition Sciences, The University of Queensland, St Lucia, QLD Australia; 6 School of Health Sciences, Swinburne University of Technology, Hawthorn, VIC Australia; 7 Physical Activity Laboratory, Baker Heart & Diabetes Institute, Melbourne, VIC Australia; 8 Institute for Physical Activity and Nutrition (IPAN), School of Exercise and Nutrition Sciences, Deakin University, Geelong, VIC Australia

**Keywords:** 24-hour physical behavior, accelerometer, measurement property, self-report assessment, time-use composition

## Abstract

**Background:**

Brief measures of 24-hour movement behaviors are needed to easily evaluate their durations. The present study investigated the criterion validity and test-retest reliability of a brief self-report instrument to assess 24-hour movement behaviors.

**Methods:**

A paper-based self-administered questionnaire was used to assess sleep, sedentary behavior (SB), light-intensity physical activity (LPA), and moderate-to-vigorous physical activity (MVPA) with four items in 35 healthy adults. Participants wore a tri-axial accelerometer and answered the questionnaire on the final day of the accelerometry assessment and after 14 days. Spearman’s correlations of self-reported measures with their accelerometer-derived counterparts were assessed and median values compared using Mann-Whitney U-tests. Bland-Altman plots were employed to characterize differences in self-reported and device-measured time in the behaviors and their limits of agreement. Test-retest reliability was assessed using Intra-class correlation coefficients (ICCs).

**Results:**

Moderate correlations with device measures for sleep, SB, and LPA for a typical and the past week (rho = 0.46 to 0.60, respectively) and low correlations for MVPA (rho = 0.33 to 0.47, respectively) were observed. Less duration of sleep and MVPA were reported compared with accelerometer-derived durations for the three recall periods (*z* = −3.9 to −2.5 and −4.0 to −3.5, respectively). The test-retest reliability for a typical week was fair-to-good or excellent for all the four behaviors (ICCs = 0.72–0.90).

**Conclusion:**

Findings show acceptable validity and reliability of this questionnaire measure of 24-hour movement behaviors for typical week, past week, and previous day recall periods.

## INTRODUCTION

Physical activity, sedentary behavior, and sleep (collectively known as ‘movement behaviors’) have all been shown to be associated with health outcomes. Engaging in at least 150 minutes per week of moderate-to-vigorous-intensity physical activity is associated with decreased risk of all-cause mortality and incident cardiovascular diseases^1^ and there are benefits of light-intensity physical activity for cardiometabolic risk factors and mortality risk.^2^ Distinct from physical inactivity, sedentary behavior, defined as any waking behavior of ≤1.5 metabolic equivalents of task (METs),^3^ is detrimentally associated with risk of all-cause, cardiovascular and cancer mortality, and incident type 2 diabetes.^4^^,^^5^ For sleep, a duration of 7 to 9 hours is generally recommended among adults for reducing all-cause mortality risk, and incidence of cardiovascular diseases and type 2 diabetes.^6^ Therefore, optimizing time spent in these behaviors can be an effective way to improve health.^7^

These movement behaviors are inherently interrelated because they are constrained within a 24-hour period. Thus, a change of one behavior results in change in the others. Concordant with this perspective, recent research has investigated relationships between movement behaviors and various health outcomes using compositional data analysis to consider each behavior as a component of 24 hours. Janssen et al^8^ reported the composition of 24-hour movement behaviors was associated with all-cause mortality, adiposity, and cardiometabolic biomarkers for adults. Moreover, replacement with moderate-to-vigorous-intensity physical activity of any other behaviors within a 24-hour period was favorably associated with these health outcomes, whereas replacement with sedentary behavior resulted in unfavorable associations. Despite the growing body of literature reporting the associations of those behaviors with health outcomes, the number of studies in large populations is still limited.^9^ Hence, further investigation in large-scale surveys is required to understand the relationship between 24-hour movement behaviors and health in the general population.

To date, 24-hour movement behaviors have been examined using data from tri-axial accelerometers or a combination of accelerometers and self-reported sleep, sedentary, and active behaviors separately.^10^ Although devices can capture time on movement behaviors with high validity,^11^ they are costly and may be burdensome for some participants.^12^ Thus, self-report instruments are required for monitoring trends and comparisons within populations over time, particularly when cost, time, or accessibility constraints limit the employment of device-based measures.^13^ In this context, a brief self-report questionnaire, which assesses time in these behaviors over the whole 24-hour period, would be useful. Given the issues of recall bias and error associated with questionnaires, examining the validity and reliability of the measure is critical to allow the collection of accurate and consistent data.^14^

According to Šuc et al,^15^ eight single questionnaires have previously been developed to capture time spent in 24-hour movement behaviors. However, most of these questionnaires do not ask responders for time spent on sedentary behavior, or do not ensure that the total time of the behaviors is equal to 24 hours, which might result in the difficulty of utilizing them for the investigation of the relationship between 24-hour movement behaviors and health outcomes. The Daily Activity Behaviors Questionnaire (DABQ) uniquely evaluates all four movement behaviors as behavioral components of 24 hours; however, time spent in light-intensity physical activity is not reported by responders but calculated by researchers in the analytic process.^16^ Previous research reported that respondents’ answers can potentially be influenced by the order of questions or context.^17^ Therefore, in the case where there is no explicit question on light-intensity physical activity, respondents might answer the questions on other behaviors (ie, sleep, sedentary behavior, and moderate-to-vigorous-intensity physical activity) without the realization that light-intensity physical activity is also a component of 24-hour movement behaviors. Also, time spent in each 24-hour movement behavior is calculated by summing times in each behavior in several domains employing 32 items. Considering low response rate has been related to increased number of questions on questionnaires,^18^ a brief questionnaire with fewer items is required.

We examined the criterion validity of a brief self-administered questionnaire assessing daily time spent in sleep, sedentary behavior, light-intensity physical activity, and moderate-to-vigorous-intensity physical activity, summing up to 24 hours (movement behaviors). Recall periods of a typical week, the past week, and the previous day were assessed separately to test whether this questionnaire would be valid for different recall periods, providing flexibility for use in wider studies. Test-retest reliability for typical week recall was also examined.

## METHODS

### Participants and data collection

Adults aged 20 to 59 years were recruited using volunteer sampling through the study coordinator (AK) in Japan in September 2023. A total of 35 adults were asked to wear an accelerometer for 7 consecutive days, and to provide socio-demographic information. On the day after the final day of accelerometry measurement (time 1) and 2 weeks later (time 2), participants answered the questionnaires on 24-hour movement behaviors including sleep, sedentary behavior, light-intensity physical activity, and moderate-to-vigorous-intensity physical activity. All the participants completed the measurements between September and October 2023. This study was approved by the Ethics Committee of Waseda University (2023-039) and written informed consent was obtained from all the participants.

### Measures

#### 24-hour movement behaviors questionnaire

A questionnaire assessing 24-hour movement behaviors, including sleep, sedentary behavior, light-intensity physical activity, and moderate-to-vigorous-intensity physical activity (see eMaterial 1 for English-language translation) was administered to participants on paper. The questionnaire was newly developed in the Japanese language through research group discussion. The primary strategy in this questionnaire was first to recall time (in hours and minutes) spent in sleep, sedentary behavior, and moderate-to-vigorous-intensity physical activity, and then the sum of these times was subtracted from 24 hours for time spent in light-intensity physical activity. Participants were asked to recall these items over three recall periods: for a typical week, the past week, and the previous day. For more information, moderate-to-vigorous-intensity physical activity was explained as activities that require moderate or higher physical effort and cause increases in breathing or heart rate, based on the validated Global Physical Activity Questionnaire (GPAQ),^19^ whereas standing and moving the body were described as examples of light-intensity physical activity. This questionnaire premised that the sum of recalled durations for four behaviors equaled 24 hours; otherwise, time spent in light-intensity physical activity was calculated by subtracting the sum of time spent in sleep, sedentary behavior, and moderate-to-vigorous-intensity physical activity from 24 hours in the data handling, complying with the strategy of this questionnaire.

#### Device-assessed physical activity, sedentary behavior, and sleep

Device-measured time spent in physical activity and sedentary behavior was evaluated using a validated tri-axial accelerometer (Active Style Pro, HJA-350IT; Omron Healthcare, Kyoto, Japan).^20^^,^^21^ This device was worn on the left side of the waist for 7 consecutive days, when participants had a typical week, except during sleep and water-based activities (eg, bathing or swimming). Although this may not have captured typical behavior over a longer period, accelerometer wear for longer periods than 7 days was considered too burdensome for participants. When they took off the accelerometer during the day, they recorded the duration and reasons for taking it off in a diary. The data were collected in 60-second epochs and classified into three different behaviors based on its METs, which were directly predicted without resource to a multiple regression model based on types of activity (ie, locomotive and household activities); sedentary behavior (≤1.5 METs), light-intensity physical activity (1.6 to 2.9 METs), and moderate-to-vigorous-intensity physical activity (≥3.0 METs).^22^ Non-wear time was defined as no acceleration signals for ≥60 consecutive minutes, allowing some signals of <1.0 MET for up to 2 minutes.^22^^,^^23^ Time for activities and non-wear time in each day were calculated by summing applicable time from midnight to the next midnight. In this study, non-wear time was used as device-measured time in sleep. Data was valid if the wearing time was ≥10 hours in a day,^24^ and participants with at least 4 valid days (including one weekend day) were included in the analysis.^25^ Average hours of valid days for each activity were used for the analysis on a typical week and the past week. For the validation of the one-day-before movement behaviors, time spent in each behavior on the final day of the accelerometry assessment was used.

#### Sociodemographic factors

Participants reported information on their sociodemographic characteristics including gender, age, height, weight, education (high school/professional school, 2-year college, or equivalent/university or graduate school), working status (yes/no), marital status (married/unmarried), living condition (living alone/living with others), and household income (<3,000,000 yen/3,000,000 to <5,000,000 yen/5,000,000 to <7,000,000 yen/7,000,000 to <10,000,000 yen/≥10,000,000 yen). Body mass index (BMI) was calculated by weight (kg) divided by height (m) squared.

### Statistical analysis

#### Validity

The correlations between questionnaire-based and device-based 24-hour movement behaviors were tested using Spearman’s correlation analysis separately for a typical week, the past week, and the previous day, as the distribution of each behavior measure was not normal. Mann-Whitney U-test was applied to compare median self-reported time spent in each behavior with their device-measured counterpart. Bland-Altman plots presented the differences between self-reported and device-measured movement behavior (y-axis), and their averages (x-axis), with the mean difference and limits of agreement (±1.96 standard deviations [SDs]) reported. Plots were conducted separately for each behavior (sleep, sedentary behavior, light-intensity physical activity, and moderate-to-vigorous-intensity physical activity) and measurement period (a typical week, the past week, and yesterday). Linear regression analysis was used to test the variability of the mean difference and limits of agreement across the average of each behavior assessed using the self-report and device-measured methods. The Spearman’s correlation coefficients (rho) were interpreted as weak (<0.3), low (0.3 to <0.5), moderate (0.5 to <0.7), strong (0.7 to <0.9), and very strong (≥0.9).^26^ All analyses were conducted using Stata version 18.0 SE (Stata Corporation, College Station, TX, USA), and a statistically significant *P*-value was set at <0.05.

#### Reliability

Test-retest reliability for the 24-hour movement behavior questionnaire on a typical week was estimated by Intra-class correlation coefficients (ICCs) with 95% confidence intervals (CIs). In this analysis, reported time spent in sleep, sedentary behavior, light-intensity physical activity, and moderate-to-vigorous-intensity physical activity at time 1 and time 2 were compared. A two-way mixed model was applied to calculate ICCs, which were interpreted as poor (<0.4), fair to good (0.4 to <0.75), and excellent (≥0.75).^27^

### Sensitivity analysis

Sensitivity analysis was performed to assess the impact of potential overestimation of device-measured sleep for the criterion when assessing the validity of the questionnaire. In the current methods, non-wear time was expected to include water-based activities as this was the reason for non-wear identified in instructions to participants. In the present study, most of the removals were due to bathing when reported by the participants who provided reasons for removal (*n* = 12). Therefore, in the sensitivity analysis, non-wear time during the daytime reported as waking hours was distributed to light-intensity physical activity. This sensitivity analysis was undertaken for all the participants with valid data.

## RESULTS

### Participant characteristics

Table 1 presents the characteristics of the overall sample (*n* = 35). Mean age and BMI were 36.7 (SD, 12.3) years and 22.9 (SD, 4.0) kg/m^2^, respectively. About a half of the participants were female (49%) and married (54%), and the majority had high level of household income (60%), completed university education (74%), lived with others (74%), and were workers (69%). The mean wearing time of accelerometers was 15.9 (SD, 1.7) hours, and only 12 participants recorded the reasons for removals of the device. The main reasons for removing the device were because of bathing and forgetting to wear after the bathing, and the mean duration of the removal was 0.6 (SD, 0.9) hours per day. The mean test-retest period was 14 days for all the participants.

**Table 1.** Characteristics of the study sample

	*N*	%
**Total**	35	100
**Gender**		
Men	18	51
Women	17	49
**Age**, years		
Mean (SD)	36.7	(12.3)
**Education**		
Graduate school or university	26	74
2-year university or college	6	17
High school	3	9
**Employment status**		
Employed	24	69
Unemployed	11	31
**Marital status**		
Married	19	54
Unmarried	16	46
**Living condition**		
Living with others	26	74
Living alone	9	26
**Household income**		
<3,000,000 yen	7	20
3,000,000 to <5,000,000 yen	3	9
5,000,000 to <7,000,000 yen	4	11
7,000,000 to <10,000,000 yen	11	31
≥10,000,000 yen	10	29
**Body mass index**, kg/m^2^		
Mean (SD)	22.9	(4.0)
**Number of valid wearing days of accelerometers**, days		
Mean (SD)	6.8	(0.7)
**Wearing time of accelerometers**, hours/day		
Mean (SD)	15.9	(1.7)

SD, standard deviation.

### Validity

The results of analyses for criterion validity are shown in Table 2. Among 35 participants, three participants with insufficient wearing time of the accelerometer on the final day were excluded in the validation for the previous day. The correlation between self-reported and device-measured duration of sleep was low to moderate for a typical week and the past week, while for the previous day this was weak. Moreover, less time in sleep was self-reported for all recall periods. The correlations between self-reported and device-measured duration of sedentary behavior and light-intensity physical activity were low to moderate for all recall periods. No significant difference between questionnaire- and device-based sedentary behavior and light-intensity physical activity was observed for all recall periods. The correlation of questionnaire- and accelerometer-derived moderate-to-vigorous-intensity physical activity was low for all recall periods. However, the correlation coefficient for the past week was not statistically significant. For all recall periods, moderate-to-vigorous-intensity physical activity was underreported.

**Table 2.** Medians and correlations of self-reported and device-measured 24-hour movement behaviors

	Difference between self-reported and device-measured duration^a^		Self-reported duration	Device-measured duration	Correlation between self-reported and device-measured duration^b^	

	*z*	*P*-value	Median (IQR)	Median (IQR)	rho (95% CI)	*P*-value
**In a typical week**						
Sleep	−3.9	<0.001	7.0 (1.5)	8.4 (2.3)	0.58 (0.38–0.79)	<0.001
SB	0.8	0.449	11.0 (6.7)	9.9 (2.1)	0.60 (0.32–0.81)	<0.001
LPA	0.6	0.541	5.0 (7.0)	4.3 (2.6)	0.57 (0.42–0.84)	<0.001
MVPA	−3.5	<0.001	1.0 (0.5)	1.3 (0.7)	0.39 (0.01–0.58)	0.023
**In the past week**						
Sleep	−3.5	<0.001	6.5 (2.0)	8.4 (2.3)	0.46 (0.21–0.69)	0.006
SB	0.5	0.634	10.0 (7.5)	9.9 (2.1)	0.54 (0.26–0.79)	<0.001
LPA	0.8	0.414	5.0 (7.0)	4.3 (2.6)	0.56 (0.44–0.86)	<0.001
MVPA	−4.0	<0.001	0.6 (1.0)	1.3 (0.7)	0.33 (−0.09 to 0.50)	0.054
**Yesterday**						
Sleep	−2.5	0.011	6.5 (1.8)	7.5 (2.4)	0.17 (−0.36 to 0.45)	0.347
SB	0.6	0.568	12.0 (8.3)	9.4 (4.1)	0.40 (0.01–0.64)	0.024
LPA	0.8	0.417	5.0 (7.0)	4.6 (3.3)	0.69 (0.58–0.89)	<0.001
MVPA	−3.5	<0.001	0.5 (1.0)	1.2 (1.1)	0.47 (0.08–0.62)	0.007

CI, confidential interval; IQR, interquartile range; LPA, light-intensity physical activity; MVPA, moderate-to-vigorous physical activity; SB, sedentary behavior.

^a^Differences were based on Mann-Whitney U-test.

^b^Correlations were based on Spearman’s analysis.

**Figure 1.**
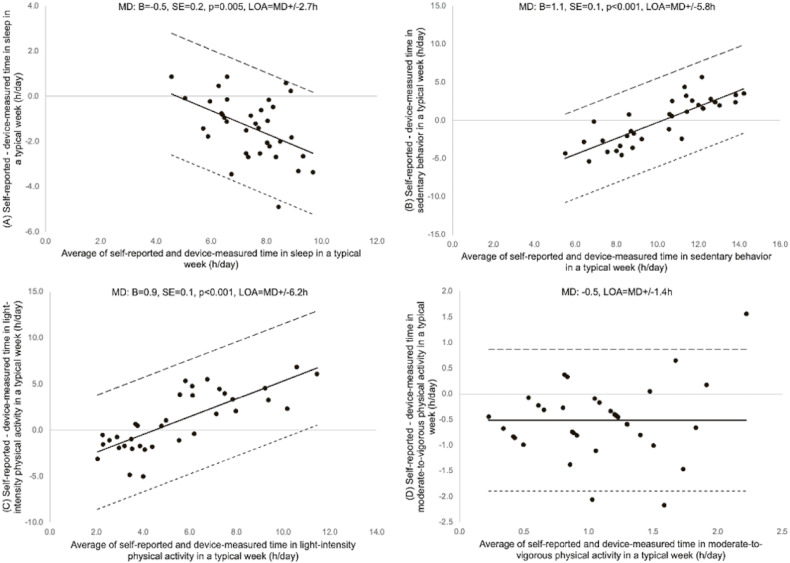
Bland-Altman plot of self-reported and device-measured (A) sleep, (B) sedentary behavior (SB), (C) light-intensity physical activity (LPA), and (D) moderate-to-vigorous physical activity (MVPA) for a typical week. The solid line represents the mean differences (MDs, hours) between two measures and the dashed lines are limits of agreement (LOA; ±1.96 standard deviations [SD]). B, partial regression coefficient; SE, standard error

**Figure 2.**
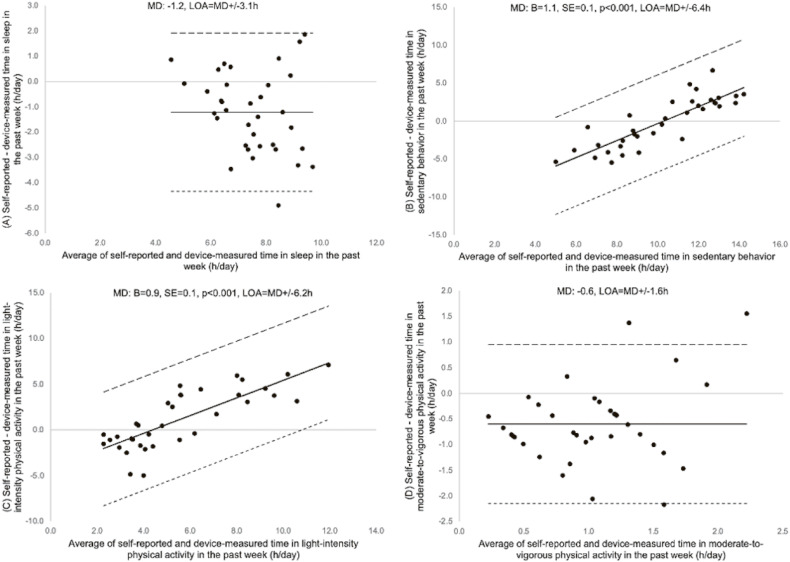
Bland-Altman plot of self-reported and device-measured (A) sleep, (B) sedentary behavior (SB), (C) light-intensity physical activity (LPA), and (D) moderate-to-vigorous physical activity (MVPA) for the past week. The solid line represents the mean differences (MDs, hours) between two measures and the dashed lines are limits of agreement (LOA; ±1.96 standard deviations [SD]). B, partial regression coefficient; SE, standard error

**Figure 3.**
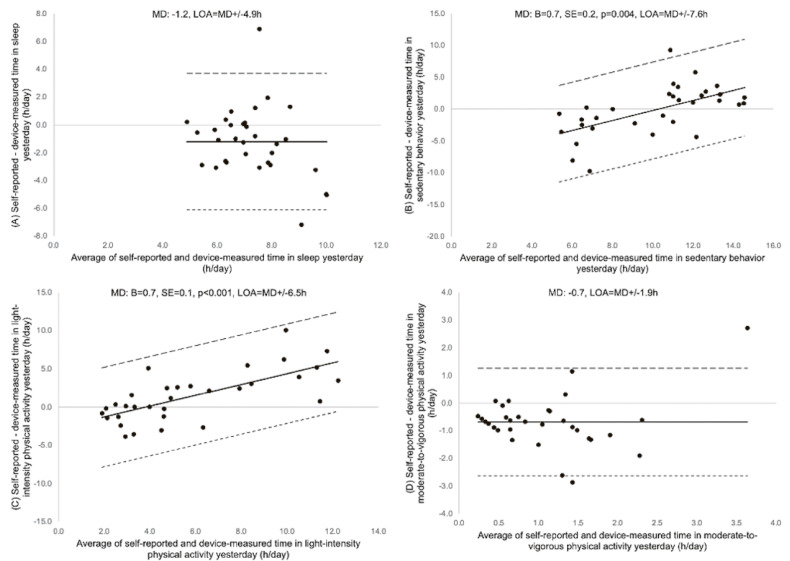
Bland-Altman plot of self-reported and device-measured (A) sleep, (B) sedentary behavior (SB), (C) light-intensity physical activity (LPA), and (D) moderate-to-vigorous physical activity (MVPA) for yesterday. The solid line represents the mean differences (MDs, hours) between two measures and the dashed lines are limits of agreement (LOA; ±1.96 standard deviations [SD]). B, partial regression coefficient; SE, standard error

The Bland-Altman plots with the agreement between the difference and the average of self-reported and device-measured 24-hour movement behaviors for a typical week, the past week, and the previous day are shown in Figure 1, Figure 2, and Figure 3, respectively. The difference was consistent across all the moderate-to-vigorous-intensity physical activity values for all recall periods, and sleep for the past week and the previous day. In contrast, linear regression showed a significant positive association for the differences and the averages of self-reported and device-measured sedentary behavior and light-intensity physical activity for all recall periods (Figure 1B, Figure 1C, Figure 2B, Figure 2C, Figure 3B, and Figure 3C), meaning greater overestimation with longer durations of sedentary behavior and light-intensity physical activity and a negative association for the difference and the average of two measures for sleep in a typical week (Figure 1A), meaning greater underestimation of sleep with longer sleep duration. The limits of agreement were ±2.7 to 4.9 for sleep, ±5.8 to 7.6 hours for sedentary behavior, ±6.2 to 6.5 hours for light-intensity physical activity, and ±1.4 to 1.9 hours for moderate-to-vigorous-intensity physical activity.

### Test-retest reliability

Table 3 shows the test-retest reliability of the 24-hour movement behavior questionnaire recalling a typical week. There was fair to good reliability between time 1 and time 2 for sleep (ICC = 0.72) and moderate-to-vigorous-intensity physical activity (ICC = 0.72) and excellent reliability for sedentary behavior (ICC = 0.90) and light-intensity physical activity (ICC = 0.88).

**Table 3.** Test-retest reliability of 24-hour movement behaviors questionnaire for a typical week

	ICC	95% CI	*P*-value	Hours at time 1	Hours at time 2

				Median (IQR)	Median (IQR)
**In a typical week**					
Sleep	0.72	0.52–0.85	<0.001	7.0 (1.5)	6.5 (1.0)
SB	0.90	0.81–0.95	<0.001	11.0 (6.7)	11.0 (6.0)
LPA	0.88	0.78–0.94	<0.001	5.0 (7.0)	5.0 (6.5)
MVPA	0.72	0.51–0.85	<0.001	1.0 (0.5)	1.0 (1.0)

CI, confidence interval; ICC, intra-class correlation coefficient; IQR, interquartile range; LPA, light-intensity physical activity; MVPA, moderate-to-vigorous physical activity; SB, sedentary behavior.

### Sensitivity analysis

The result of the sensitivity analysis is presented in eTable 1. The duration of device-based sleep decreased by 0.1 hours on the previous day and 0.6 hours for the past and a typical week compared with the main analysis, whereas that of light-intensity physical activity increased by 0.1 hours on the previous day and 1.1 hours for the past and a typical week. For the correlation of self-reported and device-derived measures, similar results were observed for all the 24-hour movement behaviors. Similar trends were shown in the differences between self-reported and device-measured durations of sleep and light-intensity physical activity. However, the differences between them for the past week were not statistically significant.

## DISCUSSION

We examined the criterion validity and test-retest reliability of a newly developed brief questionnaire for assessing 24-hour movement behaviors comprising sleep, sedentary behavior, light-intensity physical activity, and moderate-to-vigorous-intensity physical activity among healthy adults with varying recall periods. The validity of this assessment tool was shown to be acceptable for recalling a typical week, the past week, and the previous day, when compared with device-based sleep, sedentary behavior, and physical activity. Moreover, the test-retest reliability was fair-to-good or excellent for the four behaviors within 24 hours on a typical week. These findings suggest that this brief 24-hour movement behavior questionnaire could be useful as a parsimonious and practical tool to characterize time spent in sleep, sedentary behavior, light-intensity physical activity, and moderate-to-vigorous-intensity physical activity over 24 hours in a typical week, in the past week, and on the previous day among adults.

The estimates of sleep using the brief 24-hour movement behavior questionnaire showed moderate validity. The daily time spent in sleep for a typical week and the past week reported by the brief 24-hour movement behavior questionnaire had moderate correlations with non-wear time assessed using accelerometers. This result was consistent with the existing DABQ, showing strong correlations of sleep duration between this questionnaire and using accelerometers (activPAL4).^16^ Our findings differed from the DABQ regarding the differences observed for sleep. In our study, reported sleep durations were shorter than device-based non-wear time for all recall periods, whereas the DABQ found no difference of sleep duration between questionnaire and criterion measure (activPAL4). This discrepancy might be because the duration of sleep in the current study was compared with non-wear time using accelerometers, and participants took off the accelerometers while awake for bathing or other water-based activities, whereas the assessment in aquatic activities was available using activPAL4 in the previous study. Tested by assigning non-wear time during waking hours reported by participants due to bathing to light-intensity physical activity, the difference between self-reported and accelerometer-based sleep was smaller. There was no correlation between questionnaire- and accelerometer-derived sleep for the previous day recall. These findings could be due to a mismatch in how our study assessed device measured sleep (non-wearing time of accelerometers) and how participants recalled sleep time. Non-wear time was calculated as applicable time from midnight to the next midnight, whereas participants answered the questionnaire in the morning and recalled the duration after they went to bed until they woke up. Regardless of these results, the validity of sleep measures using the brief questionnaire was acceptable at least for a typical week and the past week.

The validity of sedentary behavior recall was moderately correlated with device-based sedentary behavior across all recall periods. This result was in accordance with existing questionnaires assessing 24-hour movement behaviors^16^ or sedentary behavior.^28^ Furthermore, the difference between the measures of sedentary behavior using the questionnaire and accelerometers for all recall periods was not significant. In the DABQ, sedentary behavior was underestimated by 1.6 hours; the authors proposed that this underestimation of sedentary behavior might be because of less detailed domains of sedentary behavior.^16^ However, participants in our study did not underestimate the duration of sedentary behavior, despite the brief 24-hour movement behavior questionnaire having also included only one item on sedentary behavior.

Time spent in moderate-to-vigorous-intensity physical activity estimated using the brief 24-hour movement behavior questionnaire had acceptable validity. The correlation of questionnaire-based moderate-to-vigorous-intensity physical activity with accelerometer-derived counterpart was low for all recall periods. Previous investigation on validity of the DABQ^16^ and two most common questionnaires to evaluate time spent in moderate-to-vigorous-intensity physical activity^19^^,^^29^ showed correlation coefficients of ranging 0.06 to 0.41, which were similar to our findings. The durations of moderate-to-vigorous-intensity physical activity using the brief 24-hour movement behavior questionnaire were lower than those using the device for all recall periods. Kastelic et al^16^ reported a similar finding, with time spent in moderate-to-vigorous-intensity physical activity reported on the DABQ underestimated compared with that derived from activPAL4. Using accelerometers, every activity ≥3 METs was counted as moderate-to-vigorous-intensity physical activity, whereas the questionnaire was unlikely to capture brief periods of moderate-to-vigorous-intensity physical activity, resulting in underestimation of the questionnaire. Despite these results, the validity of moderate-to-vigorous-intensity physical activity was still considered acceptable.

High validity of light-intensity physical activity was revealed for all the recall periods in this study with moderate correlations between questionnaire- and device-based measures. These findings were similar to, or exceeded, those of previous studies examining the validity of self-report assessment tools (*r* = 0.43 to 0.45).^16^^,^^30^ Previous questionnaires have used different methods to recall light-intensity physical activity either summing time spent in particular behaviors (eg, housework, standing or walking at work, transport, and self-care)^30^ or, subtracting time for other behaviors from 24 hours as in the DABQ.^16^ Thus, the method of subtracting the rest of the time from 24 hours was comparable to summing durations on the domains of light-intensity physical activity to capture time spent in light-intensity physical activity within 24-hour time constraint.

Regarding sedentary behavior and light-intensity physical activity, overestimation of duration was greater when the assessed duration was longer. Conversely, underestimation of duration was greater when the assessed duration was shorter, which was similar to what was found for the DABQ.^16^ The mean differences between self-reported and objectively measured behaviors were similar for all recall periods, suggesting that our questionnaire may be applicable for many types of recall periods. Although the limits of agreement for sedentary behavior and light-intensity physical activity were wider than those of the other behaviors, these results were comparable with previous studies investigating the validity of commonly used questionnaires, such as the GPAQ^28^ and the Sedentary Behavior and Light-Intensity Physical Activity Questionnaire.^30^ However, the limits of agreement for sedentary behavior were wider than that of independent sedentary behavior questionnaires in previous studies;^31^^–^^34^ therefore, this tool would not be recommended for individual assessments, such as in small-scale intervention trials. Since the mean differences of sedentary behavior and physical activity were smaller than the previously developed DABQ^16^ and GPAQ,^35^ the 24-hour movement behavior questionnaire tested in the present study could be considered as being useful to assess time on behaviors in large-scale studies, such as population health surveillance.

The test-retest reliability of the questionnaire for a typical week was satisfactory. The ICCs of this study were similar to or greater than previous study on the 24-hour movement behavior assessment^16^ for all the behaviors, showing the utility of the measure in studies with repeated measures.

Our study had limitations. First, self-reported sleep time was compared with non-wear time of accelerometers. Accelerometers were taken off not only in bed but also in water-based activities (ie, swimming or bathing), which can result in underestimation of device-measured sedentary behavior and physical activity and potential overestimation of sleep. Indeed, participants self-reported that they took off the device for an average of 0.6 hours per day; however, the intensity of the activity they were engaged in during non-wear was not well recorded, as only 12 participants reported the durations and reasons for the removals. To assess the potential impact of overestimation of device-measured sleep, this study undertook a sensitivity analysis, where non-wear time while participants were awake reported by them was classified into light-intensity physical activity. This classification was chosen as the main reason for removing the accelerometer was bathing. The sensitivity analysis showed similar results to the main analysis. Overestimation of device-measured sleep might still affect the estimation of sedentary behavior and light-intensity physical activity because the device might not be worn especially before and after bed even if the participants were awake. However, the device used in the present study has the advantage of more accurately detecting time in SB and PA because different equations are used in estimating METs of the signals depending on the types of activity, compared to wrist-worn accelerometers, such as ActiGraph GT3X+.^22^ Future studies could use wrist- or thigh-worn accelerometers, which may be more acceptable for 24-hour wear, to assess the validity of sleep in this questionnaire. A second limitation was that our study included a relatively small sample size. However, the number of participants was sufficient to ensure statistical power to compare measures within subjects.^36^ Third, we used a volunteer sampling method of recruitment, and the study sample was not nationally representative. Most participants had high educational attainment and household income, who might tend to be more literate and better at recall. Thus, the characteristics of the general adult population would not be represented by the present study’s participants. Therefore, further investigation would be needed for a broader range of population groups to lead to more robust evidence. Lastly, generalizability to other age groups (eg, children, youth, or older adults) was unclear, and thus, further investigation targeting other populations would be needed. Despite these limitations, our findings can contribute to understanding adults’ movement behaviors within a 24-hour period and enhance research to investigate the relationships of these behaviors with numerous health outcomes in population surveys using the compositional data analysis and subsequently help accumulate population-based evidence to inform 24-hour movement behavior guidelines.

### Conclusion

This brief self-report questionnaire on 24-hour movement behaviors had acceptable criterion validity for differing recall periods (ie, typical week, the past week, and the previous day), and test-retest reliability for a typical week among adults. The findings suggest that the tool could be useful to assess sleep, sedentary behavior, light-intensity physical activity, and moderate-to-vigorous-intensity physical activity within 24 hours, particularly in large population studies. However, future studies are needed to apply this questionnaire to different population groups.

## SUPPLEMENTARY MATERIAL

eMaterial 1. Questionnaires (English-language translation) for assessing 24-hour movement behaviors (**A**) in a typical week, (**B**) in the past week, and (**C**) on the day before

eTable 1. Sensitivity analysis for medians and correlations of self-reported and device-measured 24-hour movement behaviors
