# Supplementary material for: Validity and Reliability of a Self-administered Questionnaire for 24-hour Movement Behaviors
Source: J Epidemiol. 2026 Apr 5;36(4):123–31. doi: 10.2188/jea.JE20250185 (PMC12975771; doi:10.2188/jea.JE20250185)
Supplement: Supplementary file 1 [file je-36-123-s001.pdf]

eMaterial 1. Questionnaires (English-language translation) for assessing 24-hour movement behaviors (**A**) in a typical week, (**B**) in the past week, and (**C**) on the day before

(A) How much time per day do you spend on the following activities in a typical week? Please calculate the total time for each activity. The total amount should equal 24 hours.

1. Sleep (not including lying down while awake) hour(s) minute(s)

2. Sitting (including lying down) hour(s) minute(s)

3. Moderate-to-vigorous physical activity*[1] hour(s) minute(s)
   *[1] Activities that require moderate or higher physical effort and cause increases in breathing or heart rate

4. Light-intensity physical activity*[2] hour(s) minute(s)
   *[2] Example.) standing and moving the body
   Please calculate the duration for the light-intensity physical activity by using the following formula: 24 hours - [(1) + (2) + (3)]

Total 24 hours 00 minute

(B) How much time per day did you spend on the following activities in the past week? Please calculate the total time for each activity. The total amount should equal 24 hours.

1. Sleep (not including lying down while awake) hour(s) minute(s)

2. Sitting (including lying down) hour(s) minute(s)

3. Moderate-to-vigorous physical activity*[1] hour(s) minute(s)
   *[1] Activities that require moderate or higher physical effort and cause increases in breathing or heart rate

4. Light-intensity physical activity*[2] hour(s) minute(s)
   *[2] Example.) standing and moving the body
   Please calculate the duration for the light-intensity physical activity by using the following formula: 24 hours - [(1) + (2) + (3)]

Total 24 hours 00 minute

(C) How much time per day did you spend on the following activities yesterday? Please calculate the total time for each activity. The total amount should equal 24 hours.

1. Sleep (not including lying down while awake) hour(s) minute(s)

2. Sitting (including lying down) hour(s) minute(s)

3. Moderate-to-vigorous physical activity*[1] hour(s) minute(s)
   *[1] Activities that require moderate or higher physical effort and cause increases in breathing or heart rate

4. Light-intensity physical activity*[2] hour(s) minute(s)

*[2] Example.) standing and moving the body

Please calculate the duration for the light-intensity physical activity by using the following formula: 24 hours - [(1) + (2) + (3)]

Total 24 hours 00 minute

eTable 1. Sensitivity analysis for medians and correlations of self-reported and device-measured 24-hour movement behaviors

	Difference between self-reported and device-measured duration [a]		Self-reported duration	Device-measured duration	Correlation between self-reported and device-measured duration [b]	
	z	p-value	Median (IQR)	Median (IQR)	rho (95% CI)	p-value
**In a typical week**						
Sleep	-2.0	0.042	7.0 (1.5)	7.8 (2.2)	0.52 (0.31–0.74)	0.002
SB	0.8	0.449	11.0 (6.7)	9.9 (2.1)	0.60 (0.32–0.81)	<0.001
LPA	0.0	0.991	5.0 (7.0)	5.4 (3.1)	0.51 (0.29–0.79)	0.002
MVPA	-3.5	<0.001	1.0 (0.5)	1.3 (0.7)	0.39 (0.01–0.58)	0.023
**In the past week**						
Sleep	-1.7	0.085	6.5 (2.0)	7.8 (2.2)	0.47 (0.22–0.67)	0.004
SB	0.5	0.634	10.0 (7.5)	9.9 (2.1)	0.54 (0.26–0.79)	<0.001
LPA	0.2	0.865	5.0 (7.0)	5.4 (3.1)	0.49 (0.32–0.79)	0.003
MVPA	-4.0	<0.001	0.6 (1.0)	1.3 (0.7)	0.33 (-0.09 to 0.50)	0.054
**Yesterday**						
Sleep	-2.0	0.044	6.5 (1.8)	7.4 (2.3)	0.16 (-0.28 to 0.48)	0.392
SB	0.6	0.568	12.0 (8.3)	9.4 (4.1)	0.40 (0.06–0.64)	0.024
LPA	0.6	0.568	5.0 (7.0)	4.7 (3.3)	0.68 (0.54–0.86)	<0.001
MVPA	-3.5	<0.001	0.5 (1.0)	1.2 (1.1)	0.47 (0.08–0.62)	0.007

CI, confidential interval; IQR, interquartile range; LPA, light-intensity physical activity; MVPA, moderate-to-vigorous physical activity; SB, sedentary behavior.
[a] Differences were based on Mann-Whitney U-test
[b] Correlations were based on Spearman's analysis
